# Towards a Consensus View on Understanding Nanomaterials Hazards and Managing Exposure: Knowledge Gaps and Recommendations

**DOI:** 10.3390/ma6031090

**Published:** 2013-03-20

**Authors:** Geoffrey Hunt, Iseult Lynch, Flemming Cassee, Richard D. Handy, Teresa F. Fernandes, Markus Berges, Thomas A. J. Kuhlbusch, Maria Dusinska, Michael Riediker

**Affiliations:** 1 Centre for Bioethics & Emerging Technologies, St Mary’s University College, London, TW1 4SX, UK; E-Mail: geoffrey.hunt@smuc.ac.uk; 2 Centre for BioNano Interactions, School of Chemistry and Chemical Biology, University College Dublin, Belfield, Dublin 4, UK; E-Mail: iseult.lynch@cbni.ucd.ie; 3 National Institute for Public Health and the Environment (RIVM), Bilthoven 3720 BA, The Netherlands; E-Mail: flemming.cassee@rivm.nl; 4 Institute for Risk Assessment Sciences, Utrecht University, NL-3508 TD Utrecht, The Netherlands; 5 Ecotoxicology Research and Innovation Centre, The University of Plymouth, Drake Circus, Plymouth PL4 8AA, UK; E-Mail: r.handy@plymouth.ac.uk; 6 School of Life Sciences, Heriot-Watt University, Edinburgh EH14 4AS, UK; E-Mail: t.fernandes@hw.ac.uk; 7 Institute for Occupational Safety and Health, Deutsche Gesetzliche Unfallversicherung (DGUV), Alte Heerstr 111, Sankt Augustin 53757, Germany; E-Mail: markus.berges@dguv.de; 8 Air Quality & Sustainable Nanotechnology, Institute of Energy and Environmental Technology e.V. (IUTA), D-47229 Duisburg, Germany; E-Mail: tky@iuta.de; 9 Center for Nanointegration Duisburg-Essen (CeNIDE), University Duisburg-Essen, D-47057 Duisburg, Germany; 10 Health Effects Laboratory, Environmental Chemistry Department, NILU-Norwegian Institute for Air Research, Instituttveien 18, Kjeller 2027, Norway; E-Mail: maria.dusinska@nilu.no; 11 Institute for Work and Health, Rte de la Corniche 2, Epalinges-Lausanne CH-1066, Switzerland

**Keywords:** nanomaterial characterisation, release, exposure, hazard, nanotoxicology, nanosafety, occupational health, consensus building, life cycle approach, monitoring, protocols

## Abstract

The aim of this article is to present an overview of salient issues of exposure, characterisation and hazard assessment of nanomaterials as they emerged from the consensus-building of experts undertaken within the four year European Commission coordination project NanoImpactNet. The approach adopted is to consolidate and condense the findings and problem-identification in such a way as to identify knowledge-gaps and generate a set of interim recommendations of use to industry, regulators, research bodies and funders. The categories of recommendation arising from the consensual view address: significant gaps in vital factual knowledge of exposure, characterisation and hazards; the development, dissemination and standardisation of appropriate laboratory protocols; address a wide range of technical issues in establishing an adequate risk assessment platform; the more efficient and coordinated gathering of basic data; greater inter-organisational cooperation; regulatory harmonization; the wider use of the life-cycle approaches; and the wider involvement of all stakeholders in the discussion and solution-finding efforts for nanosafety.

## 1. Introduction

The question of the safety of nanomaterials (NMs) is one of increasing urgency as these materials enter into a wider and wider range of manufacturing processes, industrial and consumer goods and waste management processes (See the nanoproducts inventories [1]). A recent consensual report of experts in the field of nanosafety identified a general lack of coherent, consistent and well-founded data, the need for realistic exposure scenarios, better established dose–response relationships, improved extrapolation from *in vitro* to *in vivo*, and the identification of the most relevant assessment parameters [2]. In particular, there is a need: (1) to understand the dynamics of biological-nanomaterial interfaces. This includes, in the determination of NM fate and behaviour, the role and importance of the corona of biomolecules that tends to form around NMs in contact with environmental or biological fluids; (2) to embark on long-term and repeated low dose studies; and (3) to gather information about NM stability and reactivity and transformation throughout the NM lifecycle [2]. On the regulatory front the existing test guidelines need to be adapted for manufactured NMs, and on the nanosafety research front more advanced statistical and computational methods are required [2].

Over its four years of activity, NanoImpactNet hosted workshops on a range of key topics and developed consensus reports on a range of topics regarding the impact of NMs on living systems, as summarised in Table 1. At the close of the project, these findings were consolidated into a consensus report and a set of recommendations tailored for the different stakeholder groups of relevance for nanosafety, including industry, funding agencies and the research community [3], which is summarised in this article. This view is an attempt to bring together the diverse findings, conclusions and opinions of a large number and variety of specialists working in many different disciplines related directly or indirectly to the environmental, health and safety aspects of NMs. Included are chemists, biologists, material scientists, physicists, toxicologists, occupational health experts, environment and ecology specialists as well as those working in regulation, insurance, technology communication and standardisation. NanoImpactNet comprised 24 European research groups and as a result of its combined activities an engaged community with over 1,000 members was created and actively participated in nanosafety-related events. Although disagreement was to be expected within this emergent community, the authors suggest that an overall narrative appears, as outlined in this article [3].

**Table 1:** Summary of the NanoImpactNet Workshops and resulting reports.

Workpackage	Workshop Title	Report Title
Human Hazard & Exposure (WP1)	Standardization of materials and protocols	Minimal analytical characterisation of engineered nanomaterials need for hazard assessment in biological matrices [4]; First approaches to standard protocols and reference materials for the assessment of potential hazards associated with nanomaterials [5]; Best practice documents for handling, testing safety
	Methods to describe and measure exposure routes/QA	Nanoparticle metrics in the air, exposure scenarios and exposure routes [6]
	Protocols for assessment of biological hazards Biological responses	Protocols for assessment of biological hazards of engineered nanomaterials [7]
	Development of strategies to assess occupational health effects	Strategies for assessing occupational health effects of engineered nanomaterials [8,9]
Environmental Hazard & exposure (WP2)	Strategies to standardize nanomaterials for environmental and ecotoxicological research	Nanomaterials for environmental studies: Classification, reference material issues, and strategies for physico-chemical characterisation [10]; Recommendations of protocols and approaches to study environmental impacts of nanomaterials [11]
	Development of standardized protocols to determine fate and behaviour in the environment	Environmental fate and behaviour of nanoparticles—beyond listing of limitations [12]
	Optimization of current standard protocols to allow assessment of nanomaterial hazard in a range of organisms	Optimization of current standard protocols to allow assessment of nanomaterial hazard in a range of organisms [13]
Life Cycle Assessment (WP3)	Life cycle assessment of nanomaterial-containing products	The importance of life cycle concepts for the development of safe nanoproducts [14]
	Risk assessment of nanomaterials	Consensus Report - Risk Assessment of Nanomaterials: *In vitro*–*in vivo* extrapolation [15]
	Impact assessment of nanomaterials	Final report with recommendations for Impact Assessment of Nanomaterials [16]

The aim here is to present this narrative on the most significant issues of exposure and hazard assessment of NMs with a view to generating interim recommendations of use to industry, regulators, research bodies and funders. 

The consensual overview is divided into considerations of exposure, hazard, characterisation and the recommendations for the different stakeholder groups. 

## 2. Exposure

The control of the potential exposure to any hazardous material in the occupational setting is crucial. In the traditional risk framework, risk management decisions concerning occupational safety and health rely on site-specific risk assessment and information about the effectiveness of available measures to mitigate exposure [17]. 

### 2.1. Methods and NM Aggregation

The current method of assessing worker exposure to airborne particles in the workplace involves measurement of the mass concentration of health-related fractions of particles in the worker’s breathing zone. The main exception to this methodology are particle-number-based metrics for exposure for fibres and for micro-organisms [18,19]. However, nanoparticles carry extremely small masses and therefore generally contribute negligibly to the integral mass concentration of the inhalable or respirable dust fraction [20]. Besides, such a measure cannot distinguish exposure to engineered NMs from background levels of similar sized anthropogenic or ambient particles. Other metrics such as particle number concentration or surface area may be better descriptors for the biological effects of nanoparticles. The issue of exposure metrics has extensively been addressed by Maynard and Aitken [21], and they conclude that effective approaches for measuring exposure to a wide range of manufactured NMs/nano-objects will require methods for measuring aerosol number, surface area and mass concentration.

The most widespread method for determining airborne sub-micron particle number concentrations as a function of particle size, *i.e.*, particle number size distribution, is based on electrical mobility analysis of the particles [22]. This technique usually comprises three main components: (1) a particle charger to charge particles predictably depending on their size; (2) a mobility analyser which classifies the particles of one polarity according to their electrical mobility; and (3) a particle counter that determines the number concentration of the mobility-classified particles. These three components are usually employed in what has become the workhorse for occupational exposure measurements: the Scanning Mobility Particle Sizer (SMPS) or the somewhat more sensitive Fast Mobility Particle Sizer (FMPS). The International Standards Organisation (ISO) standard ISO/TR 27628 further describes the available methods to measure the above mentioned metrics of nano-objects [23]. 

However, for SMPS, no standard method has been agreed upon to produce reference particle number concentration [22]. Furthermore these instruments are bulky, expensive and complicated to use, and are therefore usually only operated by research groups and not by practitioners in SMEs for example. Fortunately, smaller and even portable devices are now appearing (e.g., from the EU FP7 NanoDevice project [24]). The SMPS also gives no information on the chemical identity of the counted particles. The latter is also true for the available small portable devices like the Condensation Particle Counter (CPC), diffusion size classifiers [25] or surface area monitors. Though they give no information of the size distribution they can be used for assessing sources of NMs or the effectiveness of control measures. The state of the art method to obtain information about size and shape as well as chemical identity and state of agglomeration is the subsequent analysis by electron microscopy of a taken sample. But no standards for sampling or analyse exist yet [20]. 

One major finding of most current studies is that during the production and handling of nanoparticles the workplace concentration of particles below 100 nm is close to the background concentration in most manufacturing companies. This background aerosol consists of ubiquitous ultrafine particles from sources in or outside e.g., particles emitted from diesel engines by trucks or forklifts, welding fumes or even vacuum cleaners with electric motors close to the process. Aggregates or agglomerates above 100 nm in size are quite often detected at the workplace and correlate with operations, further complicating detection and discrimination of NMs by existing measurement devices [26]. This is in line with theoretical calculations indicating that most of the particles emitted from processes are agglomerated when reaching the exposed person [27]. 

**Recommendation 1**: Research is needed into the safety implications of aggregation and agglomeration, especially a possible ‘Trojan Horse effect’. Emitted nanoscale entities may be loosely attached to bigger agglomerates and therefore may not be detected by the measurement devices in the size range below 100 nm. These aggregates and agglomerates can be still respirable. On inhalation they may even reach the alveoli of the lungs. They may be released in their primary size range after contact with lung surfactant. Of course, we should not only be concerned about the primary size. Research is needed into the aggregation/disaggregation mechanisms involved [20]. 

### 2.2. Release of NMs (from the Product) into the Environment 

Some early studies evaluated the release of NMs into the wider environment from some selected nanomaterial-containing products during the consumption phase [28,29]. One study used life-cycle based modelling and embraced the whole life-cycle of products that contain certain NMs [30]. This study provided the first comprehensive assessment of the potential concentrations and the associated environmental risks of nano-TiO_2_, nano-Ag and carbon nanotubes (CNTs). These studies estimated concentration levels of selected NMs in different systems (aquatic, terrestrial, waste-water systems) based on the available knowledge of the total usage of products containing NMs.

While the release of NMs is obviously a precondition of downstream exposure, little has been done so far to examine such release in a rigorous and systematic manner. General processes and areas of possible release of nanoscale entities and NMs are: the production process; the handling, packaging, use and misuse of such materials; the ageing processes of NMs; and end-of-life activities such as recycling and disposal [14].

Possible release during production may occur through leaks to water and air in closed systems or open production processes. These have been studied in several European and national studies such as NANOSH, CarboSafe and NanoGEM [31]. The areas of “handling and use” and “ageing” are vast and complex. The former includes the handling of powders, diffuse emission from production plants, mechanical treatment of NMs such as sanding and drilling, abrasion during use, spraying of ‘nano’-sprays. The latter covers all processes taking place in the environment such as selective wear and tear, degradation, wash-out, and the increased brittleness of some materials.

End-of-life activities include: (1) re-use or recycling, such as disassembling, and mechanical or thermal processes like crushing, melting and torch cutting; (2) waste treatment, such as incineration; and (3) disposal, such as landfill. The release of nanoscale entities can never be excluded as a possibility, especially during high energy processes. 

**Recommendation 2**: There is a general lack of knowledge on the release of NMs. More research and development activities need to be aimed at understanding the release of NMs from processes and products during use. This applies both to standard non-nanotechnology processes and to new nanotechnology-related processes. Specifically we need: (a) detailed studies of processes including the nanoscale; (b) standardised testing for certain possibly relevant mechanisms and routes of release; (c) the derivation of quantitative information on the possible release rates; and (d) characterisation of the physical, chemical and bio-interface properties of NMs that are dependent on specific release conditions (e.g., abrasion) that is useful for safety measures. 

**Recommendation 3**: Concerning release, for safety purposes we need data on the leach rate and/or decay rate of nanoscale entities in realistic landfill conditions. We already know that free NMs can penetrate clay (typically used as a liner in landfill) and move into ground water, so the expectation is that there will be some long-term slow release at the end of a product’s life. Of course, at this early stage, if product manufacturers can design for re-use then the amount going to landfill may be reduced. 

### 2.3. Workplace Exposure Measurements

A limitation on nanosafety development is the lack of information about the health of *already* exposed and thus potentially affected populations. There was (and still is) no European system to register occupational health related to nanomaterial exposure. Consequently, occupational health reporting strategies need further exploration and harmonization [9,8]. The workplace is generally the best characterised exposure scenario due to the expected highest exposure probability and concentrations for humans. Still most of the studies conducted to date have been on general exposure or release. That is, no specific personal exposure measurements for nanoparticles at various workplaces, leading to a robust exposure assessment via inhalation or oral uptake, have been conducted so far [9,8]. 

Dermal exposure to NMs has not been studied with the exception of Van Duuren-Stuurman *et al.* [32] using a shortened version of the observational dermal exposure assessment (DREAM) to estimate the likelihood of exposure. However, the relevance of uptake via the intact skin has been demonstrated [33]. With the exception of the intended use of NMs in food, possible oral uptake following inhalation exposure has not been studied yet.

All nano-exposure studies conducted so far are related to short-term exposures. No procedure of exposure monitoring or long term exposure assessment has been conducted to the knowledge of the authors. The current workplace investigations focus on areas where NMs are initially produced. Knowledge on use and processes with NMs in the secondary or later stages cannot currently be assessed due to lack of knowledge on their use. Labelling, which is also needed to identify possible exposure via consumer goods, is one way to address this safety research topic [34].

Comparative assessments of different tasks and processes in the workplace should be based on an extensive data set generated in as harmonised a way as [35]. The data should come along with auxiliary contextual information that is required to interpret the measurement results for risk assessment and mediation purposes. The exposure scenarios are also needed to derive information on uptake for combined assessments of hazard and exposure potential.

**Recommendation 4**: We recommend the early development and testing of personal devices delivering reliable results to reduce NMs exposure in the workplace. Focus should be set to personal real-time instruments that simulate uptake, e.g., deposition in the different areas of the respiratory tract. 

### 2.4. Safe Handling of Nanomaterials 

Accountability for workplace safety is the preparedness to provide to the relevant stakeholders an adequate account of preventive procedures followed. This will depend on clear documentation of all steps involved during handling of NMs including available data on the specific materials involved, the measures taken to provide safe handling; surveys of measures taken for safe handling on a regular basis; and the introduction of a health surveillance system related to nanomaterial production and use.

The principle of protecting workers by handling procedures that prevent and minimise exposure applies also to NMs, but special account needs to be taken of specific characteristics such as their strong tendency to reduce their surface energy by agglomerating or binding to available molecules.

Safe systems of work such as those exemplified to the Control of Substances Hazardous to Health (COSHH [36]) seem appropriate for NMs. The evidence so far is that routine protective measures such as rubber gloves, the use of dust masks suitable for ultrafine materials, and safety glasses also work for NMs when modest volumes are used (such as in the research laboratory). Safety managers should not be unduly concerned that strategies or normal procedures will not work. However, the information in the public domain used for these risk assessments needs updating for NMs. For example, the information on material safety data sheets (MSDS) should be specific to the material, and not generic information for an existing chemical form of the same substance. For example, an MSDS specific for “carbon nanotubes”, not just for ‘carbon’, is required to adequately address the potential risks of these NMs.

**Recommendation 5**: The handling of NMs in powdered form should be kept to a minimum, especially for high aspect ratio NMs (e.g., nanotubes, nanorods and nanowires) and must be performed under appropriate ventilation conditions (ideally in a closed system, but at least a fume hood) and wearing appropriately refined personal protection equipment (safety glasses, mask, gloves and lab coat or protective suit). Based on current best-practice, all solid or powdered NMs need to be labelled for safe work procedures [37,38].

**Recommendation 6**: For commercially available NMs, where a nano-specific MSDS exists (e.g., created following the Swiss recommendations [39], this should be stored in a file in the laboratory or company for each nanomaterial. It is important to note however, that most nano-specific MSDS are currently incomplete and often state that no information is available regarding the hazards or exposure consequences. For laboratory synthesised samples especially there may be very little safety information available and limited information regarding the storage and handling conditions. An MSDS for the nearest available commercial nanomaterial or the base material should be obtained and stored in the laboratory, with a note attached saying that this is a material similar to that being utilised and similar conditions would apply. The same applies if the MSDS pertains to a non-nanoscale form, to the effect that there may be additional or different risks associated with the nanoscale form of the material and to apply caution when handling. 

**Recommendation 7**: Steps should be initiated where and when appropriate for an ongoing review of all workplace particulate protection equipment in the light of developing nano-hazard and safety findings, and with a view to the standardisation of relevant to nanoscale hazards. 

**Recommendation 8**: Many workers undergo annual health surveillance as part of risk management strategies. A mechanism needs to be in place so that the doctors conducting these assessments are kept up to date with adverse effects that are specific to possible NM exposure. For example, the focus on lung function tests for dusts might distract attention away from other risks such as immunological health, or cancer risks. At the earliest possible opportunity large-scale epidemiology studies and sample banks should be established as a resource for the future for re-analysis when specific nano-exposure and nano-hazard biomarkers emerge.

### 2.5. Exposure via Consumer Products

The public use of a wide range of nanotechnology-based consumer products will result in other exposure scenarios, involving for example the use, misuse and disposal of items of personal care, domestic cleaning, personal clothing, nano-coated products, food packaging and polymer containers such as bottles, pharmaceuticals and nano-electronic goods. So far, no systematic real-life consumer-related test scenarios of release of, and exposure to, NMs exist. Current knowledge is limited to selected tests conducted for a few spray and cream formulations and the like. We even lack adequate information on nanomaterial content in consumer products despite their large-scale and growing use. More detailed information on their use and application is needed to permit a better evaluation of possible exposure pathways. One of the main obstacles in studying consumer exposure is the reliable measurement of NMs in the different matrices of consumer goods.

During consumer usage, NMs are of course subjected to mechanical, thermal and other environmental stress conditions. Studies based on the characterisation of airborne particle release due to individual processes can currently be roughly classified in terms of NMs used for coatings and NMs used for composites. Coatings could be understood as a thin layer of composite material, as the engineered nanoparticles are intentionally embedded in a matrix material such as a polymer. However, for exposure studies composites and coatings cannot simply be compared and have to be analysed in different ways. Current measurement approaches may be inappropriate for this purpose. For example, the relatively long duration of the current aerosol measurement restricts the intensity of abrasion. This means that with higher abrasion intensity the coating would be worn off before the measurement finishes. Therefore only a limited and possibly futile simulation of exposure is possible. 

**Recommendation 9**: Effective strategies to overcome the above-mentioned limitations have to be developed. These may be based on testing of different release processes and realistic exposure scenarios and routes for different consumer applications (e.g., inkjet printers, PET bottles containing nano-clays, nano-silver impregnated garments) to allow the use of specific nanoscale measurement techniques and assessments. To assess the real impact of nanomaterial on the environment and human health, it is necessary to characterize the NMs and released particles, with feasible and refined techniques, once released into the sphere of human consumption.

### 2.6. Dosimetry

“Dose” in nanoparticle toxicology may be a more difficult concept to grasp than dose regarding, for example, highly soluble substances, as the dose does not always increase linearly with mass due to inherent tendency for NMs to agglomerate as a consequence of their high surface energy. Of course, the issue is also about which dose metric (metrology) do we need to apply in concentration-dose-response functions [5]. 

Dose–response relationships need to be established for different dose metrics: mass, surface area, redox activity, *etc.* Then, we need research clarity on whether we are speaking only of the biologically available dose, and of what kind of NM. Availability is, for example, affected by aggregation and disaggregation, agglomeration and de-agglomeration. Considerations involve *in vitro* assessment, validity and round robin testing, dosimetry and the relationship to *in vivo* exposure and dosimetry. This should cover exposure dose, medium dose, delivered dose, intracellular dose, subcellular dose, as well as organism dose, tissue dose, *etc.* (p85, 89 *et passim*) [2].

Concerning dose and inhalation, there are currently very few measurement techniques that simulate aspiration efficiency and the deposition in the trachea-bronchial and alveolar regions, which results in a mismatch between the concentration measured, the concentration inhaled, and the estimate of the deposited dose. To obtain health-related exposure information, modeling techniques have to be applied to the data. This lack of health-related exposure data, among other factors, makes the establishment of occupational exposure limits challenging. Clearly a number of specific recommendations for researchers and funders on the study of NMs and dose are contained in the above considerations.

**Recommendation 10**: Researchers need to be placed in a position to confirm the particular dose they are working with, in realistic exposure conditions. This includes detailed characterization of NM dispersions *in situ* under the assay conditions. Researchers need to be able to measure with determinable accuracy the internal dose in cells, as opposed to just reporting the mass of particles added to the media. The dosimetry must take account of issues such as NMs dispersion in the exposure media (which affects the available dose of NMs) and the potential for labels to leak out of labeled-NMs under biological conditions, confounding the interpretation of the uptake studies data. 

## 3. Characterisation

The role and importance of characterising NMs, not just in their pristine as-synthesised state but also as they exist *in situ* in complex biological matrices, such as cell culture media for *in vitro* studies, became more fully appreciated in the course of the project. Unfortunately this aspect is still missing from many published articles. This is in part due to the fact that consensus has not yet been reached on the minimal characterisation requirements for NMs (as powders, in simple dispersions and *in situ* as presented to living systems) and also because research that is currently published was conceived at a time before the need for good characterisation *in situ* was widely understood [4]. It would seem that which characterisation parameters are relevant and important depends on the specific NM and its context; therefore it may be a conceptual error to think there is a once-for-all basic and multi-purpose characterisation set for each “kind” of nanoparticle [40].

### 3.1. Characterising Released Nanomaterials

The major obstacle in studying NMs release, transformation and exposure is the characterisation of the particles themselves especially in complex matrices like the ecological environment or in a living organism. Even discrimination between particles by general type, such as engineered (e.g., CNTs) or natural (e.g., volcanic) or incidental (e.g., traffic pollutants), is difficult but of great importance when assessing exposure and for analyses that interface with health studies. In real-life scenarios the researcher may be presented with a “soup” of diverse nanoparticles to analyse and understand. This problem increases as the specific NMs become ever more removed from the actual source (both in time and space). 

Certainly, in a defined workplace environment specific NMs may be expected and the release and exposure can be targeted using specific search criteria and protocols, and hence it is often possible to limit resources to stipulate parameters exactly fitting the purpose. Some strategies and techniques have been developed and tested in workplaces (reviewed in Kuhlbusch *et al.* [41] However, severe limitations exist even for research purposes and the existing techniques cannot always be employed in routine workplace measurements. In the wider environment it becomes even more difficult or impossible to develop a reliable and feasible method as released NMs may undergo ageing and diverse transformations. 

The effect of such transformations—whether air, water, soil or biological media or systems—should ideally be taken into account in exposure modelling, where relevant. Examples of such changes include loss of coatings, change in coating composition, development of a “corona” (a fuzzy coating) which depends on the particle surface properties and the nature of the biomolecules and ions available in the surrounding milieu, and dissolution in liquid media. These changes are fast and dynamic and current technologies are not sufficiently well-developed to provide rapid assessments in a coherent manner. 

The main monitoring techniques currently employed do not necessarily give the kind of *in situ* characterisation needed. They are either microscopic methods for partial information on particle morphology, state of aggregation and chemical composition, or methods discriminating particles by size in relevant media (water, air, *etc.*). The latter sometimes allow subsequent separate analysis for chemical composition. Examples are an Aerosol Mass Spectrometer [42] or the Field Flow Fractionation technique coupled with mass spectrometry for liquids. Both methods provide information on particle-size dependent chemistry.

**Recommendation 11**: Given the complexity, in the short term possible key release processes should be identified for particular products containing specific and characterisable NMs along the life-cycle. This would involve the identification of possible exposure routes and uptake paths of engineered NMs during the life-cycle, fate and behaviour of such materials in the environment, with exposure concentrations, and enabling a wider risk assessment. We need predictive models on how a nanomaterial will interact with and be transformed by its surroundings through time, and how these changes may influence subsequent transport, accumulation and reactivity. Such data generation would be complex and advanced computational techniques may be necessary to handle them for meaningful NM risk management.

### 3.2. Sample Preparation

Many NMs are supplied as powders and need proper dispersion in vehicle media to allow the assessment of their human and environmental hazards [43]. The characterization of NMs in stock dispersions in ultrapure water or a physiological buffer is well-established and researchers generally agree on how to do this and what to measure. But, this agreement is harder to achieve for complex media (natural water, soil, cell culture media with serum proteins, and blood samples) because the NMs behave differently in each milieu, such as agglomeration or formation of a protein corona changing their surface reactivity and size. The addition of serum, albumin of detergents to allow a better and more stable dispersion could also modify the biological responses induced [44]. One solution to such sample preparation problems is to have a decision-tree about characterization based on what is possible in each type of media, and relevant for the exposure route and hazard end-point being assessed. 

A good dispersion without modification of NM characteristics, especially surface reactivity due to coating with proteins or other biomolecules, is necessary to mimic accurately real life exposures. This preparation of NMs should take into account the exposure routes and target organs to be studied. Thus during inhalation the nanoparticles will come into contact with lung lining fluid mainly composed of lipids, whereas ingested nanoparticles will encounter the acidic gastrointestinal fluids. Standardized dispersion protocols are established or under development to harmonize preparation methods (OECD guidelines) but their impacts on biological responses still need further investigation.

Despite considerable progress, there is continuing uncertainty around specific issues such as the role of the synthesis route on the surface composition of nominally identical NMs. For example, silica nanoparticles can be prepared by at least five different routes, utilising different catalysts, *etc.*, with the consequent emergence of subtle differences in the surface composition. Currently, researchers (and industry) do not distinguish between these as potentially different NMs, with potentially different uptake and impacts, and as such the published data are not always reliable and comparable. [45,46]

A key communication issue is that authors are not reporting characterisation *during* the exposures, either because of logistical hurdles or because the technology is simply not reliable in the media and not with a low enough detection limit.

**Recommendation 12**: We recommend that a pragmatic way forward is for manufacturers to cooperate in characterizing the relevant properties of the NMs at production (see Stone *et al.*, 2010 for further details [10]), and to then further explore a very limited set of parameters before use (or exposure), e.g., size and size distribution, zeta potential and adsorbed species in order to take into account the effects of storage and sterilization and how different environments/conditions affect the physicochemical characteristics of NMs [4].

**Recommendation 13**: Industry, regulators and standards organizations should cooperate in generating a nomenclature system for NMs that accounts for synthesis route and potentially for surface description including initiator/catalyst residues and potential surface impurities or defects. If this proves to be too challenging, perhaps because there are too many variables, the solution may be to stick with the current chemical nomenclature and additionally indicate particle size and shape [10], adding to the chemical product labelling a code number for the synthesis method. Like other chemicals they also need to list major impurities that relate to the synthesis. 

**Recommendation 14**: It is good practice for any assay to be cross-checked for interference, and the authors also emphasize that NM samples could interfere with the test assays themselves. We recommend the establishment of appropriate controls for such possible interference [7].

**Recommendation 15**: To develop robust procedures for routine testing of NMs with a high degree of credibility we make two recommendations: (1) cell lines which are readily available and traceable (*i.e.*, same source and commercially available) should be used rather than specifically isolated/modified cell lines; and (2) cell line characterization, and reporting of degree of confluence/passage number/cell cycle duration *etc.*, should be reported and included in any data for publication [5]. 

**Recommendation 16**: Nanosafety and related researchers should be required by editors to include information about characterization and sample preparation in their papers. Some journals now have this requirement.

If implemented, the above recommendations would facilitate the identification of differences in the base NMs that could be correlated with differences in their dispersion characteristics in the exposure medium and thus differences in their biological uptake and consequently differences in the observed dose-response characteristics in biological domains. 

### 3.3. Reference Materials and Batch Variability

The development of characterisation methodologies and protocols, and hazard assessment assays and protocols requires an adequate body of reference NMs, which are available for comparison for a specific trait of the material (usually only one parameter such as chemical concentration for traditional chemicals). A certified standard is a sample that is certified for one parameter within a prescribed precision or accuracy. The EC’s Joint Research Centre (JRC) Institute of Reference Materials and Measurements has developed a certified reference NM (silica) and the Institute of Consumer Health and Protections has assembled an archive of test nanomaterials, on which there is some agreement on some characterization measurements. However, in all cases to date, the characterization is in water or simple buffer rather that in the relevant biological or environmental media. The U.S. Department of Commerce’s National Institute of Standards and Technology (NIST) is also trying to produce reference materials. 

It is difficult to see how the research community will make significant progress and reach consensus regarding the health and environmental impacts of NMs until NMs can be made reproducible from batch to batch, and/or we have a good understanding of the acceptable variability in specific NMs characteristics (e.g., size, size distribution, surface charge, surface impurities, surface chemistry, *etc.*). This is information that is absolutely necessary *before* cells or living organisms experience the NMs differently, in terms of uptake, localization, degradation/ bioaccumulation and impact. 

Of course the issue of batch-to-batch variability has a different relevance for different users. For example, academic researchers at least need to know exactly what is in the batch; industrial producers cannot scale up commercial activity until batch problems are resolved; and regulatory testers need a steady supply of the *same* thing for routine testing. Clearly, adequate regulation of NMs for application will require a high degree of reproducibility between batches, so there is a driver to resolve this issue.

**Recommendation 17**: More effort and urgency should be attached to the cooperative development of reference materials, useful both to nanomaterial developers and to those examining health, safety and environmental aspects [5].

**Recommendation 18**: More cooperative effort on minimizing batch variability is needed. Researchers should report the batch number and state the measured impurities, which are usually related to the synthesis method.

### 3.4. Laboratory Protocols 

Much has been learned to date, but the variability of nanosafety related protocols (exposure, release, dosimetry, characterisation, hazard, *etc.*) renders the available information difficult to compare and interpret [47]. 

The authors have been party to an online-space for sharing research protocols with other members within the project. The aim was to enable laboratories throughout the world to easily compare their methods and subsequently develop common protocols and strategies for the safety testing of NMs. While those in the field were motivated to discuss the harmonisation of nanoscience protocols, considerable effort and time are required for such harmonisation. It was not always evident who is the right or available individual, organisation or unit to perform this task. 

It also became clear in the project that protocols often need to be adapted to deal specifically with nanoscale materials and also that protocols suitable for one type of NM may not be applicable to other types [7].

**Recommendations 19**: Funding agencies should request specific protocol proposals from those applying for funding and could also require the definitive description of protocols in the final reports of funded projects. Research proposals should explain why a particular protocol was chosen over another. The development of nanosafety science would be supported if peer-review journals require authors to be absolutely specific in their description of protocols within the materials and methods section, not accepting a simple reference to previously published protocols that were “slightly adapted”. 

**Recommendation 20**: Careful characterization of all exposures, and understanding of biokinetics and mechanisms/mode of actions, should be encouraged for proposals for academic research into nanosafety. This is because without the exposure characterization, and without understanding biodistribution and persistence and mechanisms of action, hazard data cannot be interpreted for risk assessment purposes. Standard protocols for dispersion of nanoparticles should be agreed and differentiation between intrinsic NM responses and realistic exposure scenarios (in which NMs are more likely to be present as agglomerates) should be made. 

**Recommendation 21**: For many protocols, we recommend that tracking and visualisation techniques, *in vitro* and *in vivo*, be invented, improved and fine-tuned to allow NM identification, quantification and characterisation *in situ* [7]. 

**Recommendation 22**: Overall, existing protocols are prone to artefacts and these should be well documented and publicized, so that all research publications include the control tests used to verify the applicability of the chosen assay to the particular NM being studied. Much can potentially be learned from the pharmaceutical and cosmetics industries, as well as from the current understanding of the biological hazards of ultrafine particles (transport pollution, *etc.*) [7].

## 4. Hazard

A great deal of groundwork was, and still is, necessary to make adequate human hazard assessment possible [4,13].

### 4.1. The Role of Hazard Assessment

A hazard is a potential source of harm or adverse health effect on a person or persons. From an environmental and ecosystem point of view one would include a range of significant organisms since persons may be indirectly harmed by harm to such organisms, e.g., through the food chain, such as bacteria, phytoplankton, fish and earthworms. Human related hazard assessment relies on *in vivo* (animal) as well as *in vitro* experiments, because human data (either from human bio-monitoring or from accident studies) will most likely not be available. Both *in vitro* as well as *in vivo* studies are prone to give false positive/negative results as an *in vitro* system cannot cover all aspects of ADMET (adsorption, biodistribution, metabolism, excretion and toxicity) of NMs even though cells of human origin are often used. 

Neither *in vitro* nor *in vivo* systems can follow the same internal life cycle of NMs in tissue or cell environment (different proteins and metabolisms) as could be expected in human. Thus even more with engineered NMs than with conventional chemicals relevant hazard assessment has to be established for the purposes of a specific risk management activity, since risk (particularly in relation to occupational and consumer safety and health) is the likelihood that a person may be harmed or suffer adverse health effects if *exposed* to a hazard. Additionally, sensitive group such as children or people with certain diseases such with asthma, cardiovascular diseases or individuals with higher genetically predisposed susceptibility to NMs should be also taken into consideration. 

Hazard plus exposure puts us in the arena of risk and risk management. Nanosafety is concerned with the identification of new hazards (with occupational, health and environmental implications) arising from the specific character of manufactured (or “engineered”) nanoscale entities. The central question of the emerging discipline of nanosafety is therefore: What are the *potential sources* of harm or adverse health effects on a person or persons—and more broadly on organisms ecologically intertwined with the health of humans—that are specific to manufactured nanoscale entities and the materials in which they may be “embedded”?

Although hazard assessment is logically prior to risk management, in creating a science and technology of nanosafety the researchers have to integrate across characterisation, hazard, release, exposure and risk management (which will always entail “risk perception”) [15]. Attempts must be made to bridge the traditional gap between exposure studies and hazard assessment studies (‘impact studies’), by designing an experimental strategy that would cover all these aspects. Ideally such a bridge would identify strategies for the simplification and harmonisation of experimental design whilst still capturing the major transformations undergone by NMs across their life cycle. In an integrated approach experiments would mimic more realistically the fate of NMs as they may undergo transformations from release, through exposure, to uptake and impact in biological systems.

### 4.2. Hazard and Scale

It is important to state here that while there remains considerable uncertainty in the literature regarding the hazards presented by NMs, the authors are clear that a material cannot be declared as toxic or non-toxic *prior* to actual experience (e.g., in a test) *just* because of its 1 nm to 100 nm scale. Science requires that one actually finds out through experimentation on the basis of the actual material that one is proposing to manufacture and put into use and finally recycle or dispose of. It is through the experimentation that one finds out whether there is a hazard or not and what the mechanism of that hazard may be. A lot of high quality experimentation, overcoming the many problems outlined above, is required to properly ground the science of nanosafety. 

As an analogy, consider that a boat that is 40 metres long and 10 metres wide is not “automatically” more hazardous than a boat that is 4 metres long and 1 metre wide made of the same materials and treated under exactly the same conditions. Everything depends on the behaviour of the boat under changed or changing *conditions*, in which the scale may bring with it properties that are relevant where they were not relevant before. For example, steering the bigger boat up the River Thames would (no doubt) prove to be more hazardous than steering the identical but smaller boat. On the other hand, the smaller boat may be more hazardous in a *different way*, such as under stormy conditions when it may have lower stability. 

Scale in the abstract is not relevant, but scale in specific circumstances *may* (or may not) be relevant. Ideally, one has to find out case by case. In practice, ways have to be found of grouping cases. In the case of NMs there is growing evidence, not yet sufficiently coherent for technological purposes, that a host of characteristics *may* become relevant under specific conditions, such as surface energy, shape, composition, charge, fine surface characteristics, ageing trajectory, molecular recognition, tendency to agglomerate, and so on [48].

### 4.3. Biological Interactions

Nanosafety scientists are especially interested in those conditions that may potentiate the characteristics of NMs in biological systems at any level: subcellular, cellular, tissue, organs, organism-systems (genetic, immunological, hormonal *etc.*), organisms, populations of organisms, ecosystems, and systems of ecosystems, especially as they impact on human life. This is an enormous field of potential study—in fact, potentially it is the entire biosphere [49]. Knowledge-sharing made the authors realize that nanosafety scientists must narrow down their research to what is of most concern from the point of view of gaining knowledge on hazards perceived to be priorities. 

Most research has been *in vitro*, with some *in vivo*, which inevitably raises questions of translation and generalization of results. Nanosafety specialists have also understood that real-world studies need to pay a lot more attention to long term studies, since certain hazards may only show up over a period of years or even decades. Long terms studies would include a concern for the potential for bioaccumulation, biodegradation *in situ,* synergistic effects and perhaps one day even subtle changes in cellular signaling

So far, very few laboratories or small companies have had access to all the different sophisticated techniques ideally required to perform full exposure, *in situ* characterisation and hazard assessment. It is hoped that QNano will help to rectify this situation through the provision of Transnational Access to state of the art characterization facilities for nanomaterials and training and dissemination in the need for characterization of nanomaterials in the exposure context [46]. One important recognized need is to determine the minimum number of metrics necessary to characterise nanoparticles in physiologically relevant media (e.g., cell culture media, plasma, lung surfactants, *etc.*) and how this can be mapped onto the ADMET (Absorption, Distribution, Metabolism, Excretion, and Toxicity) models.

**Recommendation 23**:

We recommend a well-funded, coordinated and systematic focus on the following NMs:

(1) a narrow range of ‘common’ NMs (TiO_2_, ZnO, Ag, Au, Si, C, nanoclays and a few more), that is related to;
(2) a narrow range of specific exposure and release scenarios (occupational inhalation, skin absorption, effluent water, waste disposal, abrasion and leaching processes and so on), in;
(3) a narrow range of specific biological systems (a few whole-organism test species, a few organ systems such a lung, heart, nervous system, a few organism-systems such as the immunological, and a few limited aspects of an ecosystem such as soil or fresh water, and so on.

### 4.4. Ecological Systems, Including Marine and Soil

Harms to ecological systems often generate over time the conditions for general harms to humans. Five years ago, assessment of the environmental hazards of NMs was in its infancy. The knowledge on NMs’ impacts on aquatic organisms was more advanced, although very many gaps still existed [50,51,52]. Most work was focussed on a few freshwater species (daphnids, some fish species and single-celled pelagic algae) and some microbial systems. Since that time this primary and exploratory research work has expanded to cover a wider range of phyla, although with variable incidence, and a wide range of endpoints. 

Only five years ago very few papers existed at the time using terrestrial organisms as test species, and this gap was also very acute regarding impacts on plants [52]. 

Discussions on the importance of characterisation of the relevant properties of NMs for environmental studies were also in very early stages. Environmental scientists were learning rapidly from human toxicologists drawing on the wealth of knowledge from the respiratory toxicology community, especially on ultrafine particles.

Exposure characterisation in the soil matrix is not a simple matter and special methods will need developing [10]. A core problem is that we cannot detect nanoparticles against the massive background of other particulates in the soil. The authors were party to the review of methods that had become available for nanoparticles characterisation in complex matrices, including soil [13]. Much progress had been made, with techniques like Scanning Electron Microscopy with an Energy Dispersive X-Ray Analyser (SEM-EDX), x-ray spectroscopy using synchrotron radiation, but also single-particle Inductively coupled plasma mass spectrometry (SP-ICP-MS) and flow field-flow fractionation coupled to ICP-MS (F-FFF-ICP-MS). 

**Recommendation 24**: Regarding soil we recommend that research priority is given to the following: development of new methods for nano-characterisation in soil and tissues (including cheaper methods for use in standard testing schemes); assessment of nanomaterial evolution in soil system; method development for better assessment of actual exposure of soil organisms to NMs; assessment of the nanomaterial characteristics driving their potential accumulation in food chains; the development of effect-markers to address mode-of-action in soil organisms; coupling of nanomaterial properties to their fate in soil, and effects in organisms (short to medium term).

Knowledge is also required on fluxes or rates instead of distribution coefficients on which models for chemicals are often dependent. Important rates include rates of sedimentation, re-suspension, bioaccumulation, deposition and run-off fluxes. More information is needed on agglomeration, behaviour of agglomerates, sinks *i.e.*, local accumulation, and understanding of what happens at the interfaces between water and sediments and water and biological surfaces.

**Recommendation 25**: There is some urgency for funded research into future cumulative impacts of NMs (especially metallic NMs) on marine phytoplankton, which are vital to life on earth since they produce about 50% of oxygen and are a major atmospheric carbon sink [53,54,55]. 

**Recommendation 26**: For the scientific design of ecotoxicology studies a multi-partner approach is essential. Even simple assays can generally not completely be addressed by a single partner. 

### 4.5. The Life Cycle Approach

The systematic and formalized investigation of the product life cycle stages (*i.e.*, design, production, transport and distribution, use and consumption, misuse, re-use, recycling and disposal) provides a holistic perspective on the hazards, risks, benefits and opportunities of nanoproducts. Even less formalized and rather qualitative life cycle concepts may uncover prospective knowledge and knowledge gaps. 

The term “life cycle assessment (LCA)” stands for a clearly defined methodological framework that has been developed in the early 1990’s as reported e.g., in the ISO 14040/14044 standards. LCA experts ideally rely on characterization factors for NMs elaborated by physicists, metrologists and toxicologists in order to be able to differentiate bulk material from nanoscale material during the LCA steps. LCA experts need knowledge on nanomaterial emissions during all product life cycle stages [14]. 

Life cycle assessment (LCA) and life cycle impact assessment (LCIA) may be used in the nanotechnology field to e.g., assess the relative environmental performance (e.g., material and energy consumption) of nanoproducts in comparison with their conventional equivalents. The complementary use of these different life cycle concepts with the current knowledge on risk assessment may provide a sound basis for informed decision-making by industry and regulators.

Only a few real risk assessment scenarios have been carried out so far for NMs [56]. A robust analysis of what would be needed so that this approach could be pursued has to be conducted to identify the main obstacles.

Sources of misunderstandings about LCA, limitations of approaches and potential ways forward have been uncovered during the project. No comprehensive Life Cycle Analysis (LCA) can currently be pursued due to a lack of information about the production of NMs and their release in different life cycle stages [14].

**Recommendation 27**: Life Cycle issues needing targeted funding include methods for trace analysis in environmental media; research on ageing NMs; bioaccumulation/biomagnification through the food-chain; and the determination of realistic exposure scenarios for real world applications in consumer products through time. In the medium term, an investigation on the “End of life” stage of existing products containing real NMs is needed. Funding should attempt to integrate *in vitro*, *ex vivo* and *in vivo* toxicology addressing exposure characterization, biokinetics and mechanisms of action, and relevant dose justification in relation to exposure assessment. 

**Recommendation 28**: A coherent approach using laboratory and field measurement strategies should be developed and tested for 2–3 selected cases (See Recommendation 23 above). A strategy for collection and dissemination of information on the form and amount of NMs incorporated into products should be implemented via a mandatory EU reporting system for products containing NMs.

**Recommendation 29**: Since** s**ome LCA relevant processes can be simulated in the laboratory we recommend that standard simulation procedures adapted to specific NMs containing products should be tested, validated, established and standardized. In order to apply LCA, and more specifically life cycle impact assessment (LCIA), there is a need for a simplified procedure to estimate the potential ecotoxic impact of released NMs for comparative purposes, where a lower data requirement would be sufficient.

**Recommendation 30**: Regulatory bodies should recommend and encourage an industrial and innovation approach by which end-of-life information must be fed into start-of life (design) information, closing the life cycle. Initial laboratory design of NMs (e.g., polymer NMs) should incorporate at this earliest stage of design and development the findings of hazard and risk research on nanoparticles, and incorporating knowledge of new materials that have been added to the manufacturing process such as nanocatalysts and congeners. This will help avoid toxicity scenarios such as those involving PCBs.

## 5. General Recommendations for Various Stakeholders

The development of innovative and enhanced materials generated by nanotechnology is a long-term enabling process that will be a major facilitator of an environmentally sustainable and competitive economy and will cut across many major industries, including basic materials, electronics, security, building and transport, medicine, pharmaceuticals, food and agriculture. Given its novelty and complexity, bold and sustained cooperation between industry, regulators, government agencies, regional and national funding bodies, academics, research institutions, standardisation organisations and the insurance industry is vital.

For regulatory and scientific purposes, the EC definition of “nanomaterial” will hopefully be the first step, in generating a panoply of harmonisation initiatives needed in nanotechnology development [57]. Such initiatives would cover CEN/ISO standards, nomenclature, protocols, reference materials, batch material uniformity, and technology governance communication, as illustrated in the preceding sections. 

### 5.1. Industry

Many industries assess the risks associated with their products, e.g., medical devices, food, pharmaceutical, cosmetics and chemical industries. It was reasonable to assume that those industries that develop nanotechnology-enabled products are conducting additional safety evaluations, despite the fact that current regulations do not oblige them to do so for materials of equivalent chemical composition to ones that are already approved in bulk-scale. However, these data, if in existence, generally do not get published in the scientific literature, and thus are not available to the wider scientific community. It would be very helpful if information about NMs that industry has found to be safe or unsafe by stated criteria were made more widely available to the scientific community. 

Industry and their research teams should provide data about production and use of NMs, together with detailed description of used materials, consistent with the legal framework on commercial confidentiality, health and safety and the environment. To this end industry needs to be incentivised to cooperate more closely with the nanosafety agenda. It should evaluate and define suitable positive and negative controls for toxicological research and for evaluating different measurement concepts and devices; report as much information as possible about the experiments and the characterisation of materials when publishing the data in scientific publications; and contribute to the harmonisation of data descriptors for pooling safety and health information in joint databases. It should play a more active role in defining research needs, and contribute to networking activities and collaborative projects; and support the definition of clear terms of reference for industry participation.

Industry data are often proprietary information and investments will need to be protected. Firms quite legitimately think hard about which partners they might share data with. However, many academics and public sector researchers believe it would be a great leap forward if industry scientists could be convinced to share their core exposure or dose-response data, for example, in public communications or peer-reviewed journals, thus enabling comparative assessments. 

We recommend that industry revise waste management practices to be more relevant to the nanoscale nature of particles, such as incinerator temperatures (e.g., for fullerenes), explosivity, *etc.* All mixed nanoparticle-biological waste must be first treated with a sodium hypochlorite solution (bleach) or similar to remove biological component. The remaining low volumes of nanoparticles should be disposed of as aqueous chemical waste, which goes for incineration. 

Meanwhile, waste management research should obtain data on the leach rate or decay rate of nanoproducts (products containing NMs) under realistic landfill conditions (See Recommendation 3 above).

### 5.2. Regulators

The current REACH guidelines do not have specific inclusions for NMs (see schemes in [58]. A pre-requisite to applying a nano-specific sub-set in the REACH strategy will only be logical if a unique sub-set of nano-specific biological effects are also identified. So far the latter has been elusive, with the toxic mechanisms for NMs (e.g., genotoxicity, respiratory toxicity, inflammation from oxidative stress, *etc.*) also well known for traditional chemicals. There may be uncertainties in some of the assumptions behind calculations in REACH for NMs, for example, not knowing if a release is linear over time or not in an exposure model. However, these sorts of problems apply to other chemicals too.

Regulatory bodies need to reconsider the appropriateness of current approaches and consider some technical modifications to hazard assessment [59,60,61]. Action is need on implementing a precise decision-tree on how to manage groups of materials through the regulatory process based on shared physico-chemical properties and types of probable biological effects. 

We recommend that regulators pay special attention to exposure and not just adverse effects when assessing risks; and *in situ* characterisation and internal exposure assessment should be part of testing strategy for hazard identification. Methods should be developed for a risk management approach to protecting workers from potential occupational exposure to carbon nanotubes and nanofibres. Currently the research about occupational exposure measurement principles appears to be quite well-funded. However, there may be large gaps regarding the assessment of *populations* of workers that could inform epidemiological research about the types of NMs, approximate levels and durations of exposure and their link to population characteristics such as job, activity and company profiles. 

We recommend that EU member states implement a mandatory system of reporting of NMs in products to include size and size distribution, composition and surface treatment or coating.

Regulatory agencies are advised to support the setting up of databases and registries—derived from cross-disciplinary searchers—that allow storing, pooling and analysing such data for purposes such as the assessment of potential health effects or the evaluation of the effectiveness of existing exposure reduction measures.

### 5.3. Environmental and Health Researchers

Environmental and health researchers and research bodies, whether in the public or private sector, are facing quite a challenge in regard to NMs, not least because some NMs may be persistent and cumulative. Currently no environmental monitoring technology is in place to allow for the monitoring of the expected increased environmental concentrations of persistent NMs. While we recommend this, it is certainly one point of future research which is currently not seen to be an easy task. We suggest that for the time being concentrations in the environment have to be predicted rather than measured.

Environmental studies have so far been limited to release-related studies such as Kaegi *et al.* [62] on the TiO_2_ wash-off from facades. The main way of assessing possible environmental concentration currently is using emission-based approaches and models such as those pursued by Gottschalk *et al.* [63] Here they use information on production rates, release fractions, assumed or based on measurement (e.g., sewage plant studies), and environmental transport to model environmental concentrations. These concentrations may be compared with environmental no-effect levels for plants, animals and humans to assess a possible risk. Still the application of the model is limited to *a priori* information which would be good to overcome in the future.

We suggest that the traditional approach for determining risk characterization ratios (EUSES) does not work, and EUSES predictions of environmental concentrations may not be applicable to nanoparticles, since nanoparticles are dispersed rather than dissolved. Suspension (and rate of dissolution) is not modelled for conventional chemicals. The tendency of nanoparticles to aggregate and agglomerate is also obviously not considered for conventional chemicals. It is unclear how to model partitioning of NMs attached to solid surfaces because the following are unknown or not included in current models: the extent/rate of dissolution; the extent or rate of agglomeration or settling; and the extent of association with sediment.

**Recommendation 31**: Distribution models must be updated to encompass NMs, and the focus should be on quantifying distributions e.g., for “suspended dissolved”. In addition there is a need to focus on obtaining the fundamental understanding of distributions. This knowledge can then be used to develop models. Urgent attention should be given to finding a replacement for the octanol-water partition coefficient (Kow). A parameter other than Kow is required for predicting solid phase-water partitioning, but more research in NM partitioning in environmental phases is needed before such replacements can be made. It is likely that the following parameters will assist in deriving the most relevant descriptors for NM distribution in the environment: size distribution, shape, coating/surface chemistry, synthesis method, state of agglomeration, shape, “pure” NMs *vs.* formulation/embedded NMs. Furthermore, affinity for adsorption of natural organic matter (NOM) and zeta potential with NOM as well as dissolution rate may be of high value. 

**Recommendation 32**: Routine methods should be developed for the quantification of NMs in tissues, both total mass concentration and particle number concentration per g of tissue. There are detection limits in environmental samples and there is a technical gap of some three orders of magnitude. Current instrumentation only works down to mg/l, but environmental concentrations are ng-µg/L. The timescale is short and medium term. This is a technical barrier to developing environmental monitoring programs at national levels. There are currently none for NMs in river water or soil. 

### 5.4. Governmental Agencies and Funders

The authors helped to identify a number of limitations of the OECD Technical Guidance Document (TGD) for Risk Assessment of Chemicals in relation to environmental exposure: a lack of information on physico-chemical properties relevant for NMs; a lack of access to information on production, emissions throughout the life-cycle, *etc.*; fundamental limitations of the various assumptions made such as Kow being a good descriptor for processes like sorption and bioconcentration; and fundamental limitations of the various models of fate and degradation of NMs since they are developed for organic chemicals and for non-particulates. We propose the development of three interacting models: one for dissolved ions, one for NMs and one for aggregates and agglomerates.

**Recommendation 33**: Government agencies fund and/or conduct more exposure monitoring at workplaces along the process of production, use and elimination and recycling in order to make more data available for risk assessors and researchers. They should set up registries of potentially exposed workers and if possible add information about exposure measurements and health assessments.

**Recommendation 34**: Specific public funding is required for the development of material-specific analytical methods, such as routine analytical methods for characterisation of NMs in complex matrices; interaction of specific NMs and cellular energy metabolism (with the generation of Reactive Oxygen Species as a side product); the establishment of clear Structure-Property Relationships which govern the interaction with, uptake by, trafficking within and response of biological systems.

**Recommendation 35**: One of the highest priorities must be funding to develop routine methods for the measurement of NMs in tissues. For example, information on tissue concentrations would be used to link cause and effect (dose-response), used to calculate key parameters in environmental risk assessments like bioconcentration factors (see Handy *et al.*, 2012 [64] for concerns regarding these tests), or be needed for the pharmacokinetics that are an absolute requirement for the registrations of new medicines. These are potential bottlenecks that can prevent a product being registered, and therefore prevent innovation. 

**Recommendation 36**: The kinetics of NMs in biological systems need funded research. Particle systems of well-defined and systematically variable physico-chemical properties should be employed. Some problems areas needing urgent study are as follows: The role of NMs and ‘Trojan horse’ uptake of contaminants (see Recommendation 1 above); and the relation between the ageing of NMs and the kinetics of NMs in biological systems. There is a need for funding of research on discrete body systems (cardiac, renal, neural, hormone system, *etc.*) where they have been given little attention so far. Regarding human related hazard assessment only data from animal studies are accepted so far but *in vitro* human models and co culture human models are crucially important to be further developed and considered in near future in combination with high throughput technologies and use of quality assurance and nano-specific reference standards following good laboratory practice.

**Recommendation 37**: Regional and global environmental agencies should consider funding in a number of areas where knowledge is negligible, such as organisms that are not standard OECD test organisms and searching for new end-points for NM interactions. Nearly all our existing knowledge comes from a few organisms used mainly in regulatory tests from freshwater. Risk assessors currently cannot construct species sensitivity distributions (no data on different species) and we are a long way from the notion of ‘protecting most of the organisms most of the time’ in ecosystems. Gaps include amphibians, birds, reptiles, and most of the marine phyla, and non-crop plants. Data on tropical ecosystems are also lacking. 

## 6. Conclusions

The four-year project NanoImpactNet made significant progress towards the building of consensus regarding approaches to nanosafety assessment, and the development of a framework for sharing information, best practice, approaches and protocols. In addition, the project experts identified significant knowledge-gaps and made a number of important recommendations for researchers, industry and funding agencies in order to rapidly progress the field. Especially recommended are that all parties involved in NMs production re-evaluate traditional methods and metrics to improve methods appropriate to exposure to nanoscale entities, and an ongoing review of all workplace particulate protection equipment. *Inter alia*, the authors recommend better characterisation of NMs at production, and approaches that integrate rather than separate exposure and toxicity for realistic modelling. Regulatory bodies should encourage an industrial and innovation approach by which mid-life and end-of-life information must be fed into start-of life (design) information, closing the life cycle of NMs. The authors offer recommendations to industry for safe handling of NMs and propose that EU member states implement a mandatory reporting of NMs in products to include size and size distribution, composition and surface treatment or coating.
